# Calculated Energy Dissipation Distribution in Air by Fast Electrons From a Gun Source^1^

**DOI:** 10.6028/jres.065A.014

**Published:** 1961-04-01

**Authors:** John E. Crew

## Abstract

Results of calculations on the energy dissipation distribution for electrons from a point collimated (gun) source in an infinite air medium are presented. The calculation has been made for a monoenergetic source of 0.4 Mev electrons. The method of moments has been employed, fitting the two spatial variables separately.

## 1. Introduction

Calculations of the energy dissipation distributions for fast electrons in infinite and homogeneous media have been reported for the simplest source geometries.^3^,^4^ The problem of electron penetration is considered to be the following: given a source of electrons in a material, calculate the energy deposited by the electrons in a small spherical volume as a function of its position in the material. Spencer has calculated the energy dissipation distributions for the plane perpendicular source and the point isotropic source,^3^,^4^ using the moment fitting technique which has proved successful in treating X-ray penetration.^5^ His calculations were made for a variety of materials and for a range of source energies from 25 kev to 10 Mev. Agreement with available experimental data has been good.

The present work is concerned with a source geometry involving two spatial variables, namely that of the point collimated (gun) source, and corresponds to the following experimental situation: A collimated beam of monoenergetic electrons originates at a point in the medium. As the electrons move about, they dissipate energy. The quantity we determine, *J*(*r*,*α*), is basically the energy dissipated in a small volume at a distance *r* from the source point, at an obliquity angle *α* with respect to the initial line of fire, and at an azimuthal angle *ϕ* relative to an arbitrary reference plane.

## 2. Calculation of the Moments

Following the notation of footnote (4), the electrons are assumed to be generated at energy *E*_0_, with residual range *r*_0_ and stopping power (−*dE*/*dr)_E_*_0_. If ***N*** electrons are produced by the source, then the energy dissipated in a small volume *dV=r*^2^ *drdϕd*(*cosα*) at the position (*r*,*α*,*ϕ*) is

$$\frac{dE}{dV}=\frac{N{\left(dE/dr\right)}_{E0}}{r_{0}^{2}}J(r,\alpha ).$$ (1)

Determination of the function *J*(*r*,*α*) is our objective. This is accomplished by fitting moments of the closely related function

$$F(r,\alpha )=2\pi {\left(r/r_{0}\right)}^{2}J(r,\alpha ).$$ (2)

*F*(*r,α*) is proportional to the energy dissipated per unit volume in a narrow ring of the material, whose symmetry axis is the initial line of fire, at a position (*r*,*α*). The moments

$$F_{n,l}=\frac{1}{r_{0}}\int_{0}^{r_{0}}{\left(r/r_{0}\right)}^{n}dr\int_{-1}^{1}d(\cos\;\alpha )P_{l}(\cos\;\alpha )F(r,\alpha ).$$ (3)

Thus for total energy production by the source *NE*_0_, the lowest order coefficient is

$$F_{0,0}=\frac{1}{r_{0}^{3}}\int J(r,\alpha )dV=\frac{E_{0}}{r_{0}{\left(dE/dr\right)}_{E0}}.$$ (4)

The *F_n,l_* may be represented as linear combinations of a set of basic coefficients

$$\begin{array}{l} I_{n,l}^{m,l_{0}}=\int_{0}^{1}dt{\left[\frac{(1+\alpha )t}{t+\alpha }\right]}^{m-n-\frac{1}{2}}\\ \;2\pi \int_{-1}^{1}dx\;x^{n}\int_{-1}^{1}d(\cos\;\alpha )P_{l}(\cos\;\alpha )I_{l_{0}}(t,\alpha ,x) \end{array}$$ (5)

obtained by solution of the differential equation

$$\begin{array}{l} -\frac{\partial I_{l_{C}}}{\partial t}+\cos\;\alpha \frac{\partial I_{l_{0}}}{\partial x}=\int_{0}^{2\pi }d\varphi^{\prime }\int_{-1}^{1}d(\cos\alpha^{\prime })S(t,\theta )\\ \{I_{l_{0}}(t,\alpha^{\prime },x)-I_{l_{0}}(t,\alpha ,x)\}+\frac{\left(l_{0}+\frac{1}{2}\right)}{2\pi }\delta (x)\delta (t-1)P_{l_{0}}(\cos\;\alpha ). \end{array}$$ (6)

This equation describes penetration by electrons originating on a plane, with intensity in different directions given by a Legendre polynomial. The parameter *t* is the remaining fraction of the electrons’ initial range, *x* is the distance from the source plane in units of the initial electron range, and the scattering kernel *S*(*t*,θ) has an energy dependence expressed by a factor [*t*(*t*+*α*)]^−1^. (See footnote [4].)

Using the stopping power representation of eq. (14) of footnote (4) together with eq. (12.19) of footnote (5), which by symmetry applies also to the case of isotropic detector and collimated source, we write

$$F_{n,l}=\frac{\left(\frac{n-l}{2}\right)!\left(\frac{n+l+1}{2}\right)!2^{n}}{(2l+1)n!\left(\frac{1}{2}\right)!}\sum_{i=1}^{4}A_{i}I_{n,0^{\left(n+i-\frac{3}{2}\right),l}}$$ (7)

where the *A_i_*’s are given in footnote (4).

Spencer has kindly made available to us tabulations of $I_{n,0}^{m,l_{0}}$ obtained with a generalized version of the machine program described in footnote (4). We have calculated from these a triangular set of *F_n,l_* values with *l*≤*n*≤12–*l*, 0≤*l*≤6. The spatial moments *F_n,l_* were used to compute a set of functions *F_l_*(*r*) by the function fitting procedure of footnote (4). These moments are listed in table 1. Computations have been carried to six digits throughout for the sake of internal consistency.

## 3. Construction of the Energy Dissipation Function

The fitting of the moments *F_n,l_* in *r* yields a set of functions

$$F_{l}(r)=2\pi \int_{-1}^{1}F(r,\alpha )P_{l}(\cos\;\alpha )d(\cos\;\alpha ).$$ (8)

The same asymptotic form for the trial function for *F_l_*(*r*) was used as for the plane perpendicular source calculation in footnotes 3 and 4. The function *F_l_*(*r*) is normalized to unity at the origin, this boundary condition being assured by choosing the trial function

$$\begin{array}{l} F_{l}(r)=\left(1-\frac{r}{r_{0}}\right)\gamma \exp[-A_{l}r/(r_{0}-r)]\\ \;+\frac{r}{r_{0}}\sum_{i}\frac{\alpha_{i}}{\beta_{i}^{l+2}}\left(1-\frac{r}{\beta_{i}r_{0}}\right)\gamma \exp[-A_{l}r/(\beta_{i}r_{0}-r)\\ \;\text{for}\;0\leq \frac{r}{r_{0}}\leq \beta_{i}\\ \;=0\;\text{for}\frac{r}{r_{0}}>1. \end{array}$$ (9)

**Figure 1:**
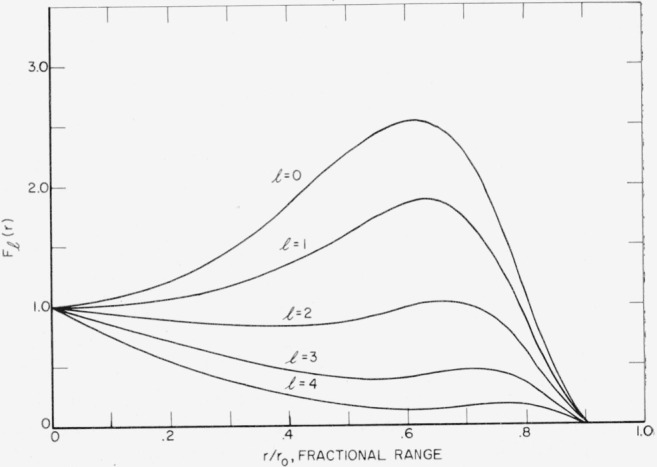
First five partial moments in r versus radial distance from the source in units of the fractional range, *r*/*r*_0_. *F_l_* (*r*) is in units of stopping power of 0.4 Mev electrons in air per unit range per electron.

The asymptotic constant *A_l_* is calculated from the two highest moments of *F_n,l_* and the coefficients *α_i_,β_i_* are computed by fitting moments according to the procedure of footnote (4). The parameter *γ* was set equal to zero for 0≤*l*≤3. The choice *γ*=1 gave a better fit for *l*=4. The results of the fitting in *r* are shown in figure 1. The results for *l*=0 correspond to the case of a point isotropic source and are in close agreement with the previous calculation by Spencer.^4^

To obtain *F*(*r,α*) it is necessary to sum the Legendre series

$$F(r,\alpha )=\sum_{l=0}^{\infty }\left(l+½\right)F_{l}(r)P_{l}(\cos\;\alpha )$$ (10)

using the set of *F_l_*(*r*)’s known for 0≤*l*≤4. However, the series has very poor convergence unless terms with *l*≫4 are included; hence it was necessary to perform an extrapolation to higher *l* values. By fitting the *F_l_* to a suitable form and making use of the generating function for Legendre polynomials, it was possible to sum the series.^6^ The following form was used over an appreciable portion of the electron range:

$$F_{l}=\sum_{i}a_{i}{\left(l+½\right)}^{n}\exp[-b_{i}(l+½)]$$ (11)

for *n* an integer. If in the generating function for Legendre polynomials

$${\left(1-2xz+z^{2}\right)}^{-1/2}=\sum_{l=0}^{\infty }z^{l}P_{l}(x)$$ (12)

the substitutions *z=e^−α^* and *x*=cos *α* are made, it is easy to show that

$$F(r,\alpha )=\sum_{i}\alpha_{i}\frac{{\left(-1\right)}^{n+1}}{\sqrt{2}}\frac{\partial^{n+1}}{\partial b_{i}^{n+1}}{\left(\cosh\;b_{i}-\cos\;\alpha \right)}^{-1/2}.$$ (13)

**Figure 2:**
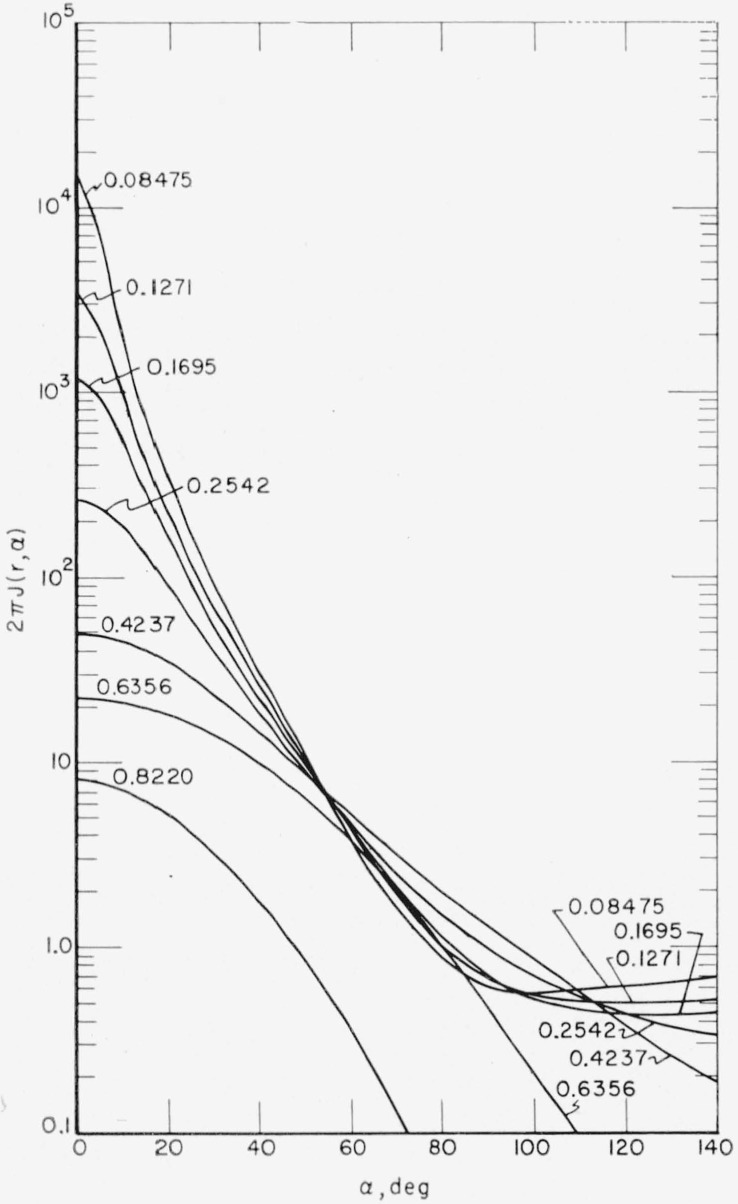
Energy dissipation function versus angle with respect to initial beam direction for various radial penetrations, *r*/*r*_0_. *J* (*r,α*) is in units of stopping power of 0.4 Mev electrons in air per sterradian per unit range per electron.

**Figure 3:**
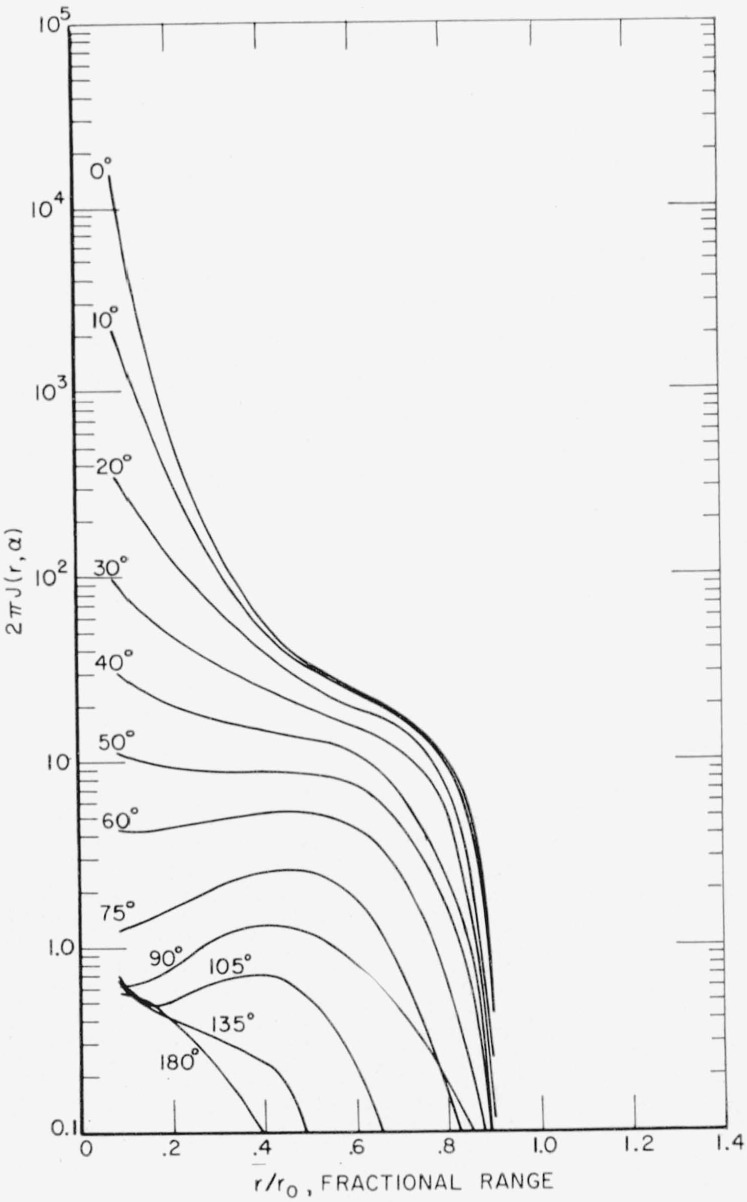
Energy dissipation function versus radial penetratration for various angles with respect to initial beam direction.

**Figure 4:**
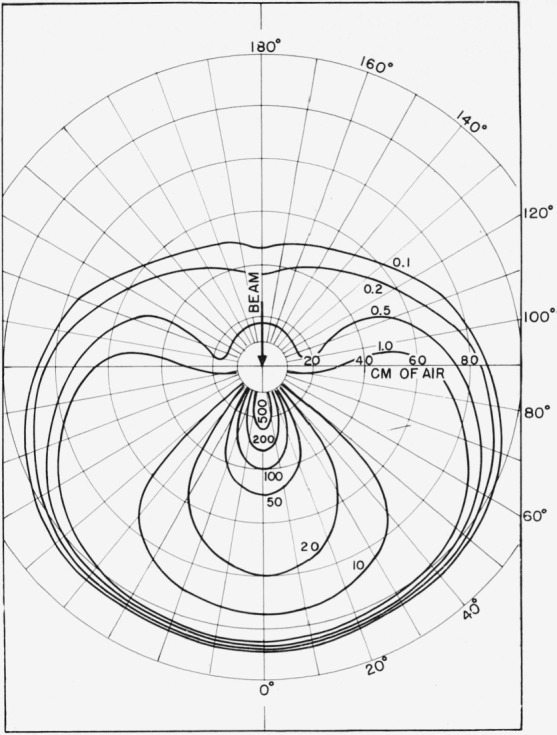
Contours of constant energy dissipation in air for 0.4 Mev electrons. Contours of 2*πJ* (*r,α*) are shown, with the radial distances giving the penetrationi n cm of air at 76 cm of Hg and 25 °C.

This method was used for *r*<0.5*r*_0_ with *n*= 1. For larger values of *r* the series converges more rapidly, so that a simpler method of extrapolating to higher *F_l_* values was used. A plot of log [*F_l_*/(*l+*1/2)*^m^*] versus *l* was made. A value of *m* yielding a straight line for the last three or four calculated *F_l_* was chosen by trial and error and the higher values obtained by extrapolating this line. The series was then summed explicitly with only a moderate number of higher *l* terms required. From *F*(*r,α*) the desired energy dissipation function *J*(*r,α*) was obtained according to eq (2). A plot of the energy dissipation function versus *α* for different penetrations *r/r*_0_ is presented in figure 2. A plot of *J*(*r,α*) versus *r/r*_0_ for different angles *α* is given in figure 3. A polar diagram showing contours of constant energy dissipation is given in figure 4.

## 4. Discussion

At present there are no experimental data with which to compare our results. Results of calculations for the one-dimensional problem have yielded results in good agreement with experiment. Calculations with slightly different trial functions generally reproduce results within about 3 percent. Since we are fitting twice, the errors can be expected to multiply, and our results cannot be expected to be better than about 10 percent. The exact value of the asymptotic constant *A_l_* is not very critical. A variation of 10 percent in *A_l_* affects the fit by more than the expected 3 percent error in fitting only on the tail of the distribution. The results in this region are not expected to be very precise anyway due to neglecting the range straggling. The values of the energy dissipation function near the source are of low precision because of the very slow convergence of the Legendre series in this region and also of low precision in the backward hemisphere for all penetrations because these values result from taking differences of nearly equal numbers.

An alternative double-fitting procedure would be to use the spatial moments in cylindrical coordinates (*z*, *ρ*). This presents an additional difficulty due to the fact that while the fitting in *z* is straightforward the asymptotic form to use in the fitting in *ρ* is not known.

## Figures and Tables

**Table 1:** Spatial moments for a point collimated source of 0.4 Mev electrons in air

The entries written as *N*(*m*) are to be interpreted as N×10*^n^*		
*n*	*l*	*F_n,l_*
		
0	0	0.143977 (1)
1	1	.512249 (0)
2	0	.383407 (0)
2	2	.176665 (0)
3	1	.182270 (0)
3	3	.558737 (−1)
4	0	.156178 (0)
4	2	.741548 (−1)
4	4	.162271 (−1)
5	1	.827478 (−1)
5	3	.265519 (−1)
5	5	.436366 (−2)
6	0	.744594 (−1)
6	2	.369759 (−1)
6	4	.854666 (−2)
6	6	.109575 (−2)
7	1	.422286 (−1)
7	3	.144003 (−1)
7	5	.251286 (−2)
7	7	.258849 (−3)
8	0	.389695 (−1)
8	2	.201798 (−1)
8	4	.500374 (−2)
8	6	.683193 (−3)
8	8	.578889 (−4)
9	1	0.232118 (−1)
9	3	.838418 (−2)
9	5	.157837 (−2)
9	7	.173430 (−3)
9	9	.123226 (−4)
10	0	.217520 (−1)
10	2	.116838 (−1)
10	4	.309866 (−2)
10	6	.457991 (−3)
10	8	.414279 (−4)
10	10	.200647 (−5)
11	1	.134557 (−1)
11	3	.511519 (−2)
11	5	.103639 (−2)
11	7	.123527 (−3)
11	9	.937227 (−5)
11	11	.539047 (−6)
12	0	.127403 (−1)
12	2	.706332 (−2)
12	4	.199041 (−2)
12	6	.317900 (−3)
12	8	.312289 (−4)
12	10	.201874 (−5)
12	12	.922263 (−7)

## Footnotes

3 L. V. Spencer, Phys. Rev. **98**, 1597, (1955).

4 L. V. Spencer, NBS Mono. **1**, (1959).

5 Fano, Spencer, and Berger, *Encyclopedia of Physics* (Springer-Verlag, Berlin, 1959) vol. **38**, pt. 2.

4 L. V. Spencer, NBS Mono 1, (1959).

